# Metabolic and Hormonal Responses to Isomaltulose Ingestion Before or During Sustained Submaximal Exercise in Adults with Type 1 Diabetes Using Automated Insulin Delivery Systems

**DOI:** 10.3390/nu16234098

**Published:** 2024-11-28

**Authors:** Olivia M. McCarthy, Merete Bechmann Christensen, Sandra Tawfik, Kasper Birch Kristensen, Bolette Hartmann, Jens Juul Holst, Signe Schmidt, Kirsten Nørgaard, Richard M. Bracken

**Affiliations:** 1 Steno Diabetes Center, Copenhagen University Hospital, Herlev, 832730 Copenhagen, Denmark; merete.bechmann.christensen@regionh.dk (M.B.C.); sandra.tawfik@regionh.dk (S.T.); kasper.birch.kristensen@regionh.dk (K.B.K.); signe.schmidt@regionh.dk (S.S.); kirsten.noergaard@regionh.dk (K.N.); 2 Applied Sport, Technology, Exercise and Medicine Research Centre, Faculty of Science and Engineering, Swansea University, Swansea SA1 8EN, UK; 3 NovoNordisk Center for Basic Metabolic Research and Department of Biochemical Sciences, University of Copenhagen, 832730 Copenhagen, Denmark; bhartmann@sund.ku.dk (B.H.); jjholst@sund.ku.dk (J.J.H.); 4 Department of Clinical Medicine, Faculty of Health and Medical Sciences, University of Copenhagen, 832730 Copenhagen, Denmark; 5 Health Technology and Solutions Interdisciplinary Research Institute, Faculty of Science and Engineering, Swansea University, Swansea SA1 8EN, UK

**Keywords:** exercise, type 1 diabetes, automated insulin delivery systems, glucose, incretin hormones, nutrition

## Abstract

Objectives: This article compares metabolic, pancreatic, and gut-derived hormone responses to isomaltulose ingestion, before versus during submaximal sustained exercise, in adults with type 1 diabetes (T1D) using automated insulin delivery systems. Methods: In a randomized, cross-over trial, eight participants with T1D being treated with automated insulin pumps (five females, age: 47 ± 16 years, BMI: 27.5 ± 3.8 kg·m^2^, diabetes duration: 23 ± 11 years, HbA1c: 8.3 ± 0.9 [67.5 ± 9.5]% [mmol/mol]) attended the laboratory on two separate occasions and consumed an isocaloric amount of isomaltulose as either (1) a single serving (0.75g CHO·kg^−1^ BM) with a 25% reduction in bolus insulin 90 min before 45 min of cycling (PEC) or (2) three separate isocaloric servings (0.25g CHO·kg^−1^ BM each) without bolus insulin during exercise (DEC). Plasma glucose (PG), gut incretins (GLP-1 and GIP), pancreatic glucagon, exogenous insulin, and whole-body fuel oxidation rates were determined. Data were treated via a two-way repeated measures ANOVA, with *p* ≤ 0.05 accepted as significant. Results: PG concentrations throughout exercise were higher and less variable with DEC compared to PEC. The exercise-induced change in PG was directionally divergent between trials (PEC: ∆ − 3.2 ± 1.2 mmol/L vs. DEC: ∆ + 1.7 ± 1.5 mmol/L, *p* < 0.001), changing at a rate of −0.07 ± 0.03 mmol/L/min with PEC and +0.04 ± 0.03 mmol/L/min with DEC (*p* < 0.001 between conditions). Throughout the exercise period, GLP-1, GIP, glucagon, and total insulin concentrations were lower with DEC (all *p* ≤ 0.02). The oxidation rates of carbohydrates were lower (*p* = 0.009) and of lipids were greater (*p* = 0.014) with DEC compared to PEC. Conclusions: The consumption of smaller servings of isomaltulose during, rather than as a single isocaloric serving before, submaximal sustained exercise provided (i) a better glycemic protective effect, (ii) a lesser push on pancreatic and gut-mediated glucoregulatory hormones, and (iii) a lower reliance on whole-body carbohydrate oxidation. Such information serves to remind us of the potential importance of nutrition for modulating the metabolic fate of an acute bout of exercise and may help inform best practice guidelines for exercise management in the T1D-sphere.

## 1. Introduction

Although endorsed for the multitude of health benefits it brings, the heightened metabolic demands of physical exercise in the context of type 1 diabetes (T1D) can cause difficulty in attaining glucose homeostasis. During exercise, the ratio of the pancreatic hormones of insulin and glucagon acts as a physiological fulcrum, balancing two ends of the metabolic spectrum to regulate the rates of glucose appearance (R_a_) versus its disappearance (R_d_), according to the requirements of working skeletal muscle tissue. Beyond the pancreatic hormones is a wider net of glucoregulatory ones that contribute to the push–pull nature of glucose control, including, but not limited to the gut-incretins, gastric inhibitory polypeptide (GIP), and glucagon-like peptide-1 (GLP-1), which are secreted after nutrient intake to facilitate R_d_ [1,2].

The implications of impaired glucoregulation in those with T1D are most evident when exercise is undertaken within the post-prandial window; hence, the risk of overt hyperinsulinemia and, thus, hypoglycemia, is expedited [3,4,5]. Accordingly, consensus guidelines governed by research studies are now inclusive of automated insulin delivery (AID) systems, advocate pre-exercise low-glycemic-index carbohydrate intake matched with reductions in bolus insulin as prudent exercise management strategies to support glycemic control [4,6,7,8,9,10,11]. However, while our understanding of how the type of carbohydrate ingested around exercise affects glucose is fairly comprehensive [9,12], far less is known about how alterations in the timing of their consumption influence glucose metabolism in those with T1D, particularly against the background of the newest generation of insulin delivery systems. The purported advantages of ingesting carbohydrates during endurance exercise have mainly been narrowed down to the associated exercise performance benefits brought about by the maintenance of blood glucose concentrations and higher rates of carbohydrate oxidation [13]. However, in the context of clinical cohorts, perhaps greater than those pertaining to sports performance are the potential benefits of carbohydrate feeding during exercise for metabolic control.

Isomaltulose is a low-glycemic-index (quantified as 32) disaccharide composed of glucose and fructose units, linked by α-1,6 glycosidic bonds [14]. By virtue of its slower digestibility and absorption via the small intestine, isomaltulose produces more stable glucose kinetics than other carbohydrate sources [9,12]. Indeed, various studies have shown that isomaltulose ingestion attenuates post-prandial insulin, glucagon, and GIP release while increasing GLP-1 secretion [15,16,17]. Yet, despite their involvement in the modulation of post-prandial glucose metabolism and, indeed, evolvement toward their use as pharmacological glucose-lowering agents for the therapeutic management of T1D, research investigating the endogenous incretin hormone responses to exercise under different feeding strategies in those with T1D is scarce. With the rapidity seen in the evolution of diabetes therapeutics and technologies comes a continued need to consider their integration around exercise, thereby informing best practice for management guidelines. Thus, this study sought to compare the glycemic, pancreatic-endocrine, and incretin hormone responses to exercise when adults with T1D who were using automated insulin delivery systems consumed a source-matched (isomaltulose) but dose-distinct (before versus during exercise) low-glycemic-index carbohydrate.

## 2. Methods and Materials

### 2.1. Study Design and Ethical Approval

**Figure 1:**
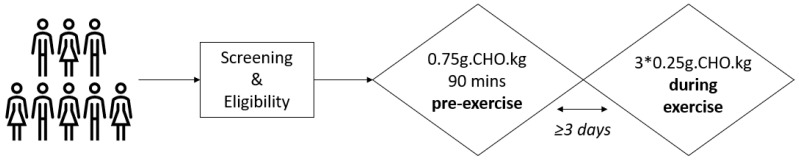
Study schematic. CHO: Carbohydrates.

This was a two-period, in-patient, randomized, crossover study involving eight adults with T1D (Figure 1).

The study was carried out in accordance with the Helsinki Declaration, the EU Directive on good clinical practice, and ICH-GCP guidelines after approval by the Regional Scientific Ethical Committee and the Capital Region’s Videnscenter for Dataanmeldelser (P-2021-169). All participants were provided with a full written and verbal description of the study and gave informed consent prior to taking part. The study was a sub-analysis of a larger randomized crossover clinical trial (IRB Trials Register: NCT05133765) [10].

### 2.2. Screening Procedures

Participants in the present study were recruited from a separate, but simultaneously conducted and ongoing randomized, crossover trial exploring the efficacy of the MiniMed 780G in people with elevated HbA_1c_ [18], during which trial the following eligibility criteria applied. Main inclusion criteria: aged 18–75 years; T1D ≥ 2 years; HbA_1c_ ≥ 7.5% (58 mmol/mol); use of insulin pump treatment for ≥12 months; use of a continuous, or an intermittently scanned, glucose monitoring system for ≥6 months; use of insulin aspart (Novo Nordisk A/S, Bagsværd, Denmark) for ≥1 week. Main exclusion criteria: females who were pregnant or breastfeeding; use of glucose-lowering medications (other than insulin), corticosteroids, and/or other drugs affecting glucose metabolism during the study period or within 30 days prior to the study starting; prior use of an AID system; daily use of acetaminophen; alcohol or drug abuse; conditions contraindicating HbA_1c_ < 7% (53 mmol/mol).

After confirmation of their suitability for the main study, participants were asked whether they would be interested in participating in the present exercise sub-study. Interested individuals then performed a graded exercise test to volitional exhaustion on a workload-controlled cycle ergometer (Corival, Lode©, Groningen, The Netherlands) as previously described [19,20]. The results were used to determine the individualized workload (watts) required to complete the moderate-intensity (~60% V̇O_2peak_) exercise sessions incorporated in each of the exercise study’s experimental visits.

### 2.3. Insulin Therapy Regime

All participants were treated with the MiniMed™ 780G system (Medtronic, Northridge, CA, USA) and had been using the device for >4 weeks prior to undertaking the exercise visits. The technology automatically adjusts basal insulin levels every 5 min based on continuous glucose monitoring input, working within adjustable glucose targets of 100 (5.5), 110 (6.1), and 120 (6.7) mg/dL (mmol/L), and includes an automatic correction bolus feature. A raised temporary glucose target of 150 (8.3) mg/dL (mmol/L) can be set for scenarios such as exercise. By doing so, the auto-correction feature is suspended, and the automatic basal insulin delivery rate is adjusted to attain the temporary target glucose level. User-initiated meal announcements are required for optimal glycemic results [11,21]. The participants used Guardian 3 link or Guardian 4 transmitters connected to the MiniMed™ 780G system and were advised to change their sensor 24 h before the trial visit if it had been used for >5 days.

### 2.4. Experimental Day Procedures

Ahead of their scheduled appointment, the participants were asked to refrain from undertaking physical exercise in the 24 h prior to each experimental visit. Verification of adherence was monitored via the International Questionnaire for Physical Activity upon arrival (PEC: 4097 ± 5382 vs. DEC: 3425 ± 4658 METS; *p* = 0.334). Participants were also asked to refrain from consuming alcohol and to be diligent in the avoidance of hypoglycemia (defined as a finger-tip blood glucose value of <3.9 mmol/L) in the 24 h prior to each visit. The participants arrived at the research facility following an overnight fast (≥10 h) from food, with water ad libitum. Upon arrival, the participants adopted a bed-rest position and were fitted with an indwelling intravenous cannula in the antecubital fossa.

Following the first sample draw (baseline), the participants consumed a standardized, low-glycemic-index, carbohydrate-based drink in a 1:10 product-to-water ratio, i.e., as a 10% concentration ([isomaltulose, BENEO GmbH, Mannhein, Germany] equating to 0.75 g of carbohydrates per kg body mass [grouped mean: 60 ± 10 g]), supplied in two different ways according to randomization: (1) as one serving given 90 min before exercise and accompanied by a 25% reduction in their usual meal time bolus insulin dose (5.2 ± 3.3 units), representing ‘pre-exercise carbohydrates’ (PEC), or (2) in three isocaloric, isovolumic servings of 0.25 g of carbohydrates per kg of body mass each (grouped mean 20 ± 3.5 g per serving), i.e., immediately before (t = 0 min), at the mid-point of (t = +20 min), and toward the end of (t = +40 min) exercise without bolus insulin, representing ‘during exercise carbohydrates’ (DEC). In the PEC group, the temporary target was applied to the automated insulin delivery system 90 min before exercise commencement [21]. In the DEC group, the temporary target was applied 15 min before exercise commencement. In both arms of the study, the temporary target stayed activated until 15 min after exercise.

Participants performed 45 min of dynamic sustained exercise (5 min warm-up, followed by 40 min of cycling at 60% V̇O_2peak_) on a workload-controlled cycle ergometer (Corival, Lode©, Groningen, The Netherlands). Breath-by-breath data were measured via indirect calorimetry (Vyntus™ ONE, Vyaire medical, Mettawa, IL, USA) with integrated heart rate monitoring (Polar Electro, Kempele, Finland). Raw cardiopulmonary data were exported at 5-s intervals (SentrySuite™ software (Version 3.20), Vyaire medical, Mettawa, IL, USA) and subsequently averaged in 30-s segments for statistical processing. The rates of carbohydrate and lipid oxidation were calculated using the principles of stoichiometry [22].

### 2.5. Blood Sampling Procedures

Venous-derived whole blood samples were obtained at baseline and then immediately before (t = 0 min), at the mid-point of (t = +20 min), and immediately after cycling (t = +45 min). The samples were centrifuged immediately to determine the point concentrations of plasma glucose (PG) and lactate (YSI Inc., Yellow Spring, OH, USA). The remaining volume was stored at −80 °C for the subsequent determination of pancreatic (insulin and glucagon) and incretin (GIP and GLP-1) hormone responses. Insulin concentrations were analyzed with a commercially available sandwich ELISA kit (Iso-Insulin ELISA, cat. no. 10-1128-01, Mercodia, Uppsala, Sweden). Plasma concentrations of total GIP and total GLP-1 were measured with commercially available sandwich ELISA kits (cat. nos: 10-1258-01 and 10-1278-01, Mercodia, Uppsala, Sweden). The detection limit for the GIP ELISA is 1.62 pmol/L and for the GLP-1 ELISA, the detection limit is 0.65 pmol/L. Plasma was extracted in a final concentration of 70% ethanol before glucagon analysis. Glucagon content was measured using a C-terminally directed antiserum (code no. 4305), measuring the glucagon of pancreatic origin as described previously [23]. Sensitivity for the assay was below 1 pmol/L, and the intra-assay coefficient of variation was below 10%.

### 2.6. Participant Characteristics

Baseline anthropometric, diabetes, and physical fitness characteristics of the eight study participants (five females) are displayed in Table 1.

### 2.7. Statistical Analyses

All statistical analyses were performed via IBM^®^ SPSS^®^ Statistics (Version 29.0.1.0, IBM Corp, Armonk, NY, USA). Descriptive statistics are presented as mean ± SD in the text and tables and mean ± SEM in the graphics. A two-way repeated measures ANOVA was run to determine conditional differences in the hormonal and metabolic parameters between and within the trial arms. In instances where the sphericity was violated and the Greenhouse–Geisser ε value > 0.750, Huynh–Feldt results were reported. Post hoc analyses to determine the simple effects were conducted with the Bonferroni test to account for multiple comparisons over time. Due to appreciable data loss (<10%), insulin values were handled using mean imputation. Between-trial differences in single-point values, e.g., cardiorespiratory parameters, were analyzed via paired-samples *t*-tests or, failing the assumption of normality, the non-parametric equivalent (the Wilcoxon signed-rank test). The McNemar test was used to determine differences between dichotomous dependent variables. Time spent with the PG within a specific glucose zone was calculated as the number of PG readings that fell within that zone, divided by the total number of PG readings from the participant and represented as a percentage, i.e., the time below range ([TBR] < 3.9 mmol/L [<70 mg/dL]), time in range ([TIR] 3.9–10.0 mmol/L [70–180 mg/dL]), and time above range ([TAR] > 10.0 mmol/L [>180 mg/dL]) [24]. Statistical significance was accepted when *p* ≤ 0.05.

## 3. Results

### 3.1. Plasma Glucose Responses

Table 2 displays the glycemic parameters between the trial arms overall (baseline to +45 min) and during an isolated in-exercise period (0 to +45 min).

There were identical baseline (fasting) PG concentrations (PEC: 7.6 ± 1.8 vs. DEC: 7.4 ± 1.5 mmol/L, *p* = 0.783). In neither trial arm were supplementary carbohydrates needed in order to reach the threshold set as being ‘safe to start’ exercise (PG > 5.0 mmol/L 15 min before the anticipated start time).

Three out of the eight participants commenced exercise above the recommended target range (>10 mmol/L) in the PEC group, whereas everyone started within the euglycemic range in the DEC group (*p* = 0.250).

**Figure 2:**
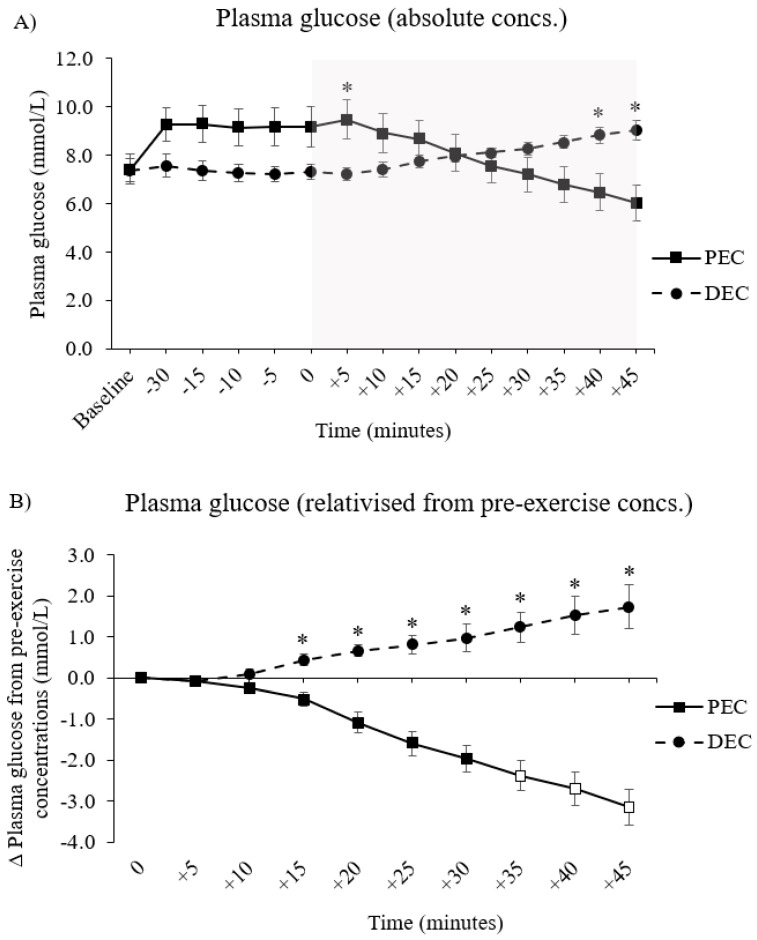
Plasma glucose responses across the experimental trial visits; data are expressed as (**A**) the absolute concentrations across the entire trial arm, i.e., inclusive of the period before exercise, and (**B**) the relativized change from the respective concentrations immediately pre-exercise (minute 0) in each arm. PEC indicates the trial arm in which the carbohydrate solution was consumed as one full serving, equating to 0.75 g/kg/bm, 90 min prior to exercise commencement. DEC indicates the trial arm in which the carbohydrate solution was consumed in three isocaloric, isovolumic servings at the start (0 min), mid-point (+20 min), and toward the end (+40 min) of exercise. * Denotes a significant difference (*p* ≤ 0.05) in the point concentration of a parameter between the two trial arms. White markers denote a significant difference (*p* ≤ 0.05) in the point concentration of a parameter, relative to the respective trial arm’s baseline value. The gray-shaded area denotes the time frame in which participants undertook a 45-min bout of moderate-intensity continuous exercise, programmed to be at ≈60% of their individualized V̇O_2peak_. Data are presented as the mean ± SEM.

A significant condition by trial interaction effect was found in PG when this variable was analyzed across the entire trial period (F (14, 84) = 25.575, *p* < 0.001 [Figure 2, panel A]). The PG trajectories were diametric between arms throughout the trial day, with point concentrations being initially higher in the PEC group during the pre-exercise period before switching to DEC throughout the exercise period (between-conditions differences in absolute PG concentration are denoted by * in Figure 2, panel A).

When data were expressed as a relativized change from pre-exercise concentrations, a significant condition by trial interaction effect was found (F (9, 54) = 41.049, *p* < 0.001). Indeed, the exercise-induced change in PG differed between the trial arms at every time point from 15 min of cycling onward (denoted by * in Figure 2, panel B; all values are *p* ≤ 0.01).

Relative to those concentrations measured immediately at the start of exercise, the PG concentrations had decreased significantly by the final 15 min of cycling in the PEC group, i.e., at minute 35 (∆ − 2.4 ± 1.1 mmol/L, *p* = 0.051), 40 (∆ − 2.8 ± 1.2 mmol/L, *p* = 0.041), and 45 (∆ − 3.2 ± 1.3 mmol/L, *p* = 0.028). Within-condition changes from the pre-exercise values are denoted by the white blocked markers in Figure 2, panel B. Conversely, there were no changes in PG from pre-exercise concentrations at any time point during exercise in the DEC group.

Everyone participant experienced a drop in PG over exercise in PEC whereas only one participant did so in the DEC group (by −0.25 mmol/L) (between-group difference, *p* = 0.016). The mean exercise-induced change in PG was significantly different between the trials (PEC: ∆ − 3.2 ± 1.2 mmol/L vs. DEC: ∆ + 1.7 ± 1.5 mmol/L, *p* < 0.001), decreasing at a rate of −0.07 ± 0.03 mmol/L/min in the PEC group and increasing at a rate of +0.04 ± 0.03 mmol/L/min in the DEC group (*p* < 0.001 between conditions).

One individual experienced hypoglycemia in the PEC group at the very end of exercise (minute 45), while there were no incidences in the DEC group (*p* = 1.00). No one experienced severe hyperglycemia (>13.9 mmol/L) in either group.

### 3.2. Plasma Lactate Responses

**Figure 3:**
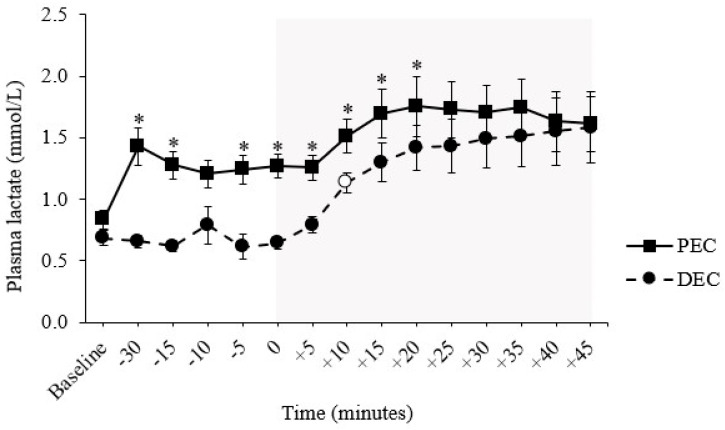
Plasma lactate responses across each experimental trial arm. PEC refers to the trial arm in which the carbohydrate solution was consumed as one full serving, equating to 0.75g/kg/bm, 90 min prior to exercise commencement. DEC refers to the trial arm in which the carbohydrate solution was consumed in three isocaloric, isovolumic servings at the start (0 min), mid-point (+20 min), and toward the end (+40 min) of exercise. * Denotes a significant difference (*p* ≤ 0.05) in the point concentration of a parameter between the two trial arms. White markers denote a significant difference (*p* ≤ 0.05) in the point concentration of a parameter relative to the respective trial arm baseline value. The gray-shaded area denotes the time frame in which participants undertook a 45-min bout of moderate-intensity continuous exercise, programmed to be at ≈60% of their individualized V̇O_2peak_. Data are presented as the mean ± SEM.

A significant condition by trial interaction effect was found (F (14, 84) = 2.067, *p* = 0.022). Plasma lactate concentrations were proportionately higher in the PEC group at almost all time points until the mid-point of the exercise session (Figure 3). In neither trial did exercise, per se, evoke a change in plasma lactate, i.e., all values remained comparable to those at the start of exercise.

Trial-day plasma lactate concentrations were moderately and positively correlated with those of both GLP-1 (r(*n* = 63) = 0.530, *p* < 0.001) and GIP (r(63) = 0.370, *p* = 0.003).

### 3.3. Incretin Hormone Responses

#### 3.3.1. Glucagon-like Peptide-1 (GLP-1)

A significant condition by trial interaction effect was found (F (1.140, 6.841) = 5.300, *p* = 0.053). Point concentrations of GLP-1 were higher in the PEC group, both immediately before (+14.1 ± 4.71 pM, *p* < 0.001, and halfway through cycling (Mid-Ex + 11.41 ± 6.74 pM, *p* = 0.004).

Relative to its individualized trial-arm baseline value, GLP-1 concentrations had risen significantly by the pre-exercise time point in the PEC group (+14.34 ± 9.58 pM, *p* = 0.045), but showed no indication of changes in any capacity throughout the exercise period. In the DEC group, concentrations of GLP-1 had risen from the baseline by the mid-exercise point (+2.31 ± 1.21, *p* = 0.014) and post-exercise point (+7.77 ± 5.35 pM, *p* = 0.051).

#### 3.3.2. Glucose-Dependent Insulinotropic Polypeptide (GIP)

**Figure 4:**
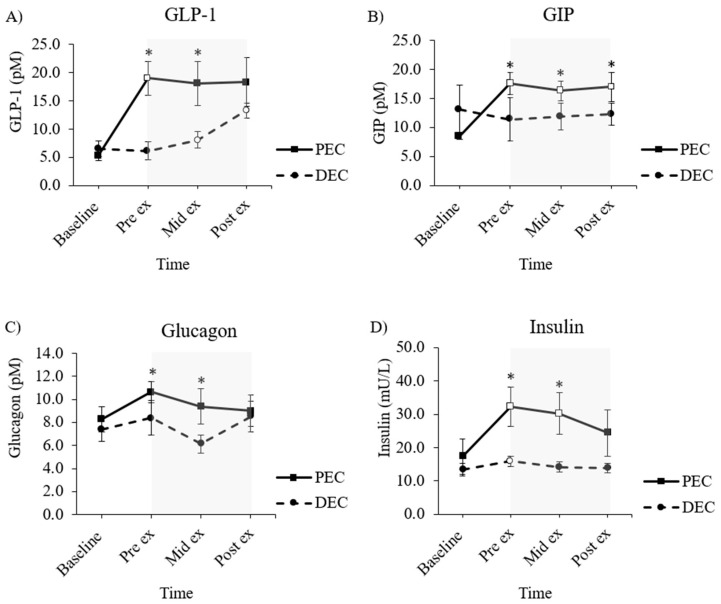
Gut peptide (**A**,**B**) and pancreatic (**C**,**D**) hormone responses across each experimental trial visit. PEC refers to the trial arm in which the carbohydrate solution was consumed as one full serving, equating to 0.75 g·kg·bm, 90 min prior to exercise commencement. DEC refers to the trial arm in which the carbohydrate solution was consumed in three isocaloric, isovolumic servings at the start (0 min), mid-point (+20 min), and toward the end (+40 min) of exercise. * Denotes a significant difference (*p* ≤ 0.05) in the point concentration of a parameter between the two trial arms. White markers denote a significant difference (*p* ≤ 0.05) in the point concentration of a parameter relative to the respective trial arm baseline value. The gray-shaded area denotes the time frame in which participants undertook the 45-min bout of moderate-intensity continuous exercise, programmed to be at ≈60% of their individualized V̇O_2peak_. Data are presented as the mean ± SEM.

A significant condition by trial interaction effect was found (F (1.799, 10.794) = 11.884, *p* = 0.002). GIP concentrations were significantly higher in the PEC group at all time points following the baseline (all *p* < 0.01 [* Figure 4, panel B]). Compared to its respective baseline values, the PEC group was associated with increases in GIP across all time points, i.e., pre-exercise (∆ + 8.90 ± 5.38 pM, *p* = 0.028), mid-exercise (∆ + 7.97 ± 4.29 pM, *p* = 0.016) and post-exercise (∆ + 8.44 ± 5.75 pM, *p* = 0.049), but with no evidence of any exercise-induced changes. There were no intra-trial changes in the DEC group of any nature.

### 3.4. Pancreatic Hormone Responses

#### 3.4.1. Glucagon

There was no significant condition by trial interaction effect (F (3, 15) = 0.631, *p* = 0.606). Point concentrations of glucagon were notably higher in the PEC group, both immediately before (+3.17 ± 1.83 pM, *p* = 0.008) and at the mid-point of (+3.17 ± 2.14 pM, *p* = 0.015) exercise. In neither trial did glucagon levels change from their respective baseline concentrations, nor were there any indications of an exercise-induced rise in glucagon over the 45-min cycling bout.

#### 3.4.2. Insulin

A significant condition by trial interaction effect was found (F (1.857,12.998) = 6.434, *p* = 0.013). Insulin concentrations were significantly higher in the PEC group at exercise onset (+16.33 ± 15.71 μL, *p* = 0.022) and at the mid-point of cycling (+16.00 ± 14.75 μL, *p* = 0.018), but had begun to homogenize by cycling cessation (+10.56 ± 14.8 μL, *p* = 0.084).

Relativized to the individualized trial-arm baseline concentrations, insulin levels were raised at both the start (+14.88 ± 10.76 μL, *p* = 0.035) and mid-point of cycling (+12.80 ± 8.26 μL, *p* = 0.019), with a trend for continued elevation upon exercise cessation (+7.06 ± 5.66 μL, *p* = 0.058) in the PEC group. In the DEC group, only pre-exercise insulin concentrations were raised from baseline (+2.57 ± 1.01 μL, *p* = 0.001).

### 3.5. Exercise Physiological Responses

Table 3 displays the cardiorespiratory parameters collected from the 45-min bout of moderate-intensity continuous exercise at a fixed workload, set to achieve a ~60% V̇O_2peak._

The DEC group was associated with a lower RER and decreased rates of carbohydrate oxidation, along with a greater percentage contribution of lipids to the total energy yield.

## 4. Discussion

The aim of this study was to compare the glycemic, pancreatic-endocrine, and incretin hormone responses to exercise when adults with T1D who are using automated insulin delivery systems consumed a source-matched (isomaltulose) but dose-distinct (before versus during exercise) low-glycemic-index carbohydrate. Our findings revealed that apportioning smaller amounts of a low-glycemic-index carbohydrate during, rather than a singular serving before, exercise provided (i) a better glycemic protective effect, (ii) a lesser push on pancreatic and gut-mediated glucoregulatory hormones, and (iii) a lower reliance on whole-body carbohydrate oxidation.

### 4.1. Glycemic Responses

Guidance for performing post-prandial physical exercise in those with T1D advocates the consumption of carbohydrates alongside a reduced dose of bolus insulin in a pre-planned fashion, ahead of the intended activity [4,6,25]. Despite integrating these guidelines in the present study, the inherent need for rapid-acting insulin, even a reduced dose, to manage post-prandial glycemia after consuming the larger single serving of carbohydrates (60 g; ≈240 kcal) in the PEC arm resulted in a two-fold higher background of circulating insulin concentrations at exercise onset than those measured in the DEC group. Although the plasma glucose levels immediately prior to starting exercise were similar between the groups following the two feeding strategies, the synergistic and, indeed, additive glucose-lowering effects of peripheral hyperinsulinemia and working skeletal muscle tissue actions in the PEC group likely promoted an increased rate of intramuscular glucose uptake over the 45-min cycling session (regression analyses, with circulating insulin at exercise onset and the Δ in glucose over exercise β = −0.603, *p* = 0.013). The result of this was a precipitous drop (~ −3 mmol/L) in plasma glucose over exercise, a trend that was not only completely avoided but was also directionally opposite in the DEC arm, wherein the source-matched carbohydrate was distributed as three ‘mini’-servings during exercise (≈20 g servings).

Interestingly, the PEC group’s insulin levels mimicked those of a clamp study wherein high insulin rates (≈20 uU/mL during 60 min of exercise [40% V̇O_2max_]) were infused to understand the impact of pharmacologically controlling hormones during exercise in healthy individuals [26]. In accordance with our findings, they, too, saw a substantial fall in glucose after exercise, combined with a failure to rebound in recovery. Our DEC arm reflected those findings, identified in the same study’s low insulin rate (≈9.5 uU/mL), where the drop in glucose was far less severe and did not change from the basal concentrations. Hence, the inexercise feeding strategy employed in DEC, which negated the need to inject exogenous meal-time insulin and circumnavigated the glycemic implications of the overt hyperinsulinemia that often accompanies post-prandially performed exercise in those with T1D. A novel aspect of our study cohort is that all participants were using an automated insulin delivery system (MiniMed 780G); hence, the applicability of these findings to alternative insulin therapy regimes such as multiple daily injections and/or other AID systems under different levels of background exogenous insulin is unclear.

Notably, every participant experienced a decline in glucose over exercise in PEC, whilst this was only evident for one person in DEC (of only −0.3 mmol/L, *p* = 0.016). Noteworthily, DEC was associated with over 95% time in range throughout cycling coupled with a complete avoidance of time spent in hypoglycemia. Though in this instance these metrics did not breach ‘statistical’ sig-nificance, the clinical meaningfulness of them is not entirely redundant. 

### 4.2. Glucoregulatory Hormone Responses

The ergometer cycling session in this study was submaximal in intensity (~60%V̇O_2peak_) and was sustained over a 45-min period at a fixed mechanical load. Although this form of exercise induces a low to moderate impact on several glucoregulatory hormones, e.g., catecholamines [27], it constitutes a useful model by which to explore other metabolic and hormonal biomarkers. A consequence of consuming a larger, single serving of carbohydrates in the PEC group resulted in a stimulatory effect on endogenous incretin hormones ahead of and toward the front end of exercise in our study participants. As the exercise duration progressed, the deterred and developmental supply of carbohydrates in the DEC group led to a small but progressive rise in incretins to values not significantly different from those of the PEC group by cycling cessation. This rightward shift in incretin responsivity speaks to the sensitivity of these hormones to nutrient ingestion per se. Indeed, endogenous incretins are key modulators of energy homeostasis after nutrient intake, due to their stimulatory effect on pancreatic insulin secretion [28,29]. Although, admittedly, the pancreatic islet β-cell reserve is minimal in people with long-established T1D, the elevated incretin hormone profiles in the PEC group, particularly when combined with higher background levels of exogenous insulin, may have contributed to the associated reduction in glucose levels observed during exercise.

GIP also exerts a stimulatory effect on α-mediated glucagon secretion, which may, at least in part, explain the concomitant raised glucagon concentrations noted in the PEC arm [30]. Although more work is needed, these findings help explain the role of the incretin pathway in managing a 60-g carbohydrate load of isomaltulose, given as either a single bolus before exercise or when apportioned into ‘mini’ doses that were split evenly throughout the exercise period in people with T1D.

### 4.3. Shifts in Fuel Metabolism

The submaximal, 45-min cycling session in this study required an energetic ‘cost’ of ≈900 kJ (≈200 kcals), ≈60% of which was derived from the oxidation of carbohydrates. This amounts to between 25 and 30 g of carbohydrates provided from a mixture of intramuscular and hepatic glycogen, circulating plasma glucose, and ingested exogenous carbohydrates (comprising two monosaccharides, glucose and fructose). As mentioned, the carbohydrate source used in this study was isomaltulose, a disaccharide composed of glucose and fructose units linked by α-1,6 glycosidic bonds [14], given as a 10% solution mixed with water. Similar to previous research [9,12], we observed raised lactate profiles following the ingestion of isomaltulose, a phenomenon that was independent of the effects of exercise per se. As fructose bypasses the phosphofructokinase regulatory point in glycolysis, there is an increased flux through the glycolytic pathway, which promotes the formation and subsequent release of lactate [31]. This might explain the elevated blood lactate curve in the acute post-prandial window in the PEC group, the magnitude of which waned toward homogeneity between the trials, along with the progressive provision of isomaltulose in the DEC group. Nevertheless, the small blood lactate responses to exercise (<2 mmol/L) seen in both arms demonstrate the low requirement from non-oxidative carbohydrate combustion.

An interesting finding in our data is the approximate 10% lower oxidation of carbohydrate in favor of a shift toward greater lipid oxidation during the cycling test in the DEC arm. Even small increases in insulin before exercise, as a product of high carbohydrate intake, can exert a suppressive effect on peripheral lipolysis and, hence, limit fat oxidation. Thus, the lower insulin concentrations seen throughout exercise in the DEC arm may feed into the observed differences in substrate utilization between the trials. Other research groups have noted increases in endogenous lipid combustion following the pre-exercise ingestion of a low- relative to a high-glycemic-index carbohydrate comparator [12]. The extension of these findings through alterations in the dosing schedule in the present study provides a novel insight as to the influence of time, rather than the type, of carbohydrate ingestion on whole-body fuel use.

## 5. Concluding Remarks

This study has revealed interesting insights into the impact of low-glycemic-index carbohydrates taken during rather than before exercise in modulating the glycemic, wider metabolic, and hormonal responses to submaximal exercise in individuals with T1D. A simple adjustment in the manner by which source-matched carbohydrates were consumed resulted in considerable and clinically relevant differences in several physiological responses to exercise. Such information serves to remind us of the potential importance of nutrition in modulating metabolism during an acute bout of exercise and may help inform best-practice guidelines for exercise management in the T1D sphere.

## 6. Study Strengths, Limitations, and Future Research Directions

Although the current data pertain only to moderate-intensity continuous exercise undertaken with an automated insulin delivery system, these data may help provide a foundational basis from which patients and their healthcare providers can formulate prudent exercise management strategies aimed at maximizing safety and minimizing glycemic disturbance. Although an inherent limitation of this pilot study is the small sample size, with homogeneity in diabetes and physical fitness characteristics, we hope that this information serves to help guide decision-making in clinical care. The expansion of this work to a larger, more heterogenous population, along with an investigation of possible sex differences and/or influences, as well as testing the effects of variations in feeding strategies on a wider panel of physiological parameters during exercise models that differ in intensity and/or duration should be considered in future research.

## Figures and Tables

**Table 1:** Baseline anthropometric, diabetes, and physical fitness characteristics of the study participants. BMI: body mass index. HbA1c: hemoglobin A1C (taken at the screening visit, prior to initiating automated insulin delivery treatment). TDD: Total Daily Dose of Insulin. BP: blood pressure. MAP: mean arterial pressure. HDL: high-density lipoprotein. LDL: low-density lipoprotein. VLDL: very low-density lipoprotein. V̇O_2peak_: The O_2_ uptake obtained in the 30 s prior to volitional exhaustion. Power_peak_: The workload obtained in the 30 s prior to volitional exhaustion.

Characteristic	Mean ± SD
Age (years)	47 ± 16
BMI (kg/m^2^)	27.5 ± 3.8
Diabetes duration (years)	23 ± 11
Age of diabetes onset (years)	25 ± 14
HbA1c (% [mmol/mol])	8.3 ± 0.9 (67.5 ± 9.5)
TDD (U/kg)	0.6 ± 0.1
Systolic BP (mmHg)	133 ± 12
Diastolic BP (mmHg)	77 ± 10
MAP (mmHg)	96 ± 8
Total cholestrol (mmol/L)	4.4 ± 0.7
HDL (mmol/L)	1.9 ± 0.6
LDL (mmol/L)	2.0 ± 0.5
VLDL (mmol/L)	0.5 ± 0.2
Triglycerides (mmol/L)	1.0 ± 0.4
V̇O_2peak_ (L/min [mL/min/kg])	1.9 ± 0.8 (24.2 ± 8.5)
Power_peak_ (Watts [Watts/kg])	187.5 ± 62.3 (2.4 ± 0.7)

**Table 2:** Glycemic parameters during each pre-defined time period for the two experimental trial arms.

Parameter	PEC	DEC	*p*-Value
Overall (Baseline to +45 min)			
Mean (mmol/L)	8.2 ± 2.0	7.8 ± 0.7	0.687
SD (mmol/L)	1.3 ± 0.4	0.8 ± 0.4	0.040
CV (%)	16.0 ± 4.5	10.0 ± 5.0	0.059
Max (mmol/L)	9.8 ± 2.0	9.2 ± 1.1	0.515
Min (mmol/L)	5.8 ± 1.5	6.8 ± 0.9	0.168
TBR (%)	0.9 ± 2.5	0.0 ± 0.0	0.351
TIR (%)	73.3 ± 36.8	95.8 ± 6.1	0.136
TAR (%)	25.8 ± 37.4	4.2 ± 6.1	0.155
Exercise (0 min to +45 min)			
Mean (mmol/L)	7.8 ± 2.1	8.1 ± 0.6	0.751
SD (mmol/L)	1.2 ± 0.4	0.7 ± 0.5	0.127
CV (%)	15.3 ± 4.9	8.9 ± 5.7	0.069
Max (mmol/L)	9.2 ± 2.3	9.1 ± 1.0	0.923
Min (mmol/L)	6.0 ± 2.0	7.1 ± 0.8	0.243
TBR (%)	1.4 ± 3.9	0.0 ± 0.0	0.351
TIR (%)	76.1 ± 35.4	96.3 ± 7.4	0.182
TAR (%)	22.5 ± 36.2	3.8 ± 7.4	0.221

PEC refers to the carbohydrate solution being consumed as one full serving, equating to 0.75 g/kg/bm, 90 min prior to exercise commencement. DEC refers to the carbohydrate solution being consumed in three isocaloric, isovolumic servings at the start (0 min), mid-point (+20 min), and toward the end (+40 min) of exercise. Data are presented as mean ± SD. SD: Standard deviation. Max: Mean maximum plasma glucose concentrations. Min: Mean minimum plasma glucose concentrations. CV: Coefficient of variation. TBR: Time spent with plasma glucose below the target range (<3.9 mmol/L). TIR: time spent with plasma glucose levels within the target range (3.9–10.0 mmol/L). TAR: time spent with plasma glucose above the target range (>10.0 mmol/L).

**Table 3:** Cardiorespiratory parameters collected from the 45-min bout of standardized moderate-intensity continuous exercise at a fixed workload, set to achieve a ~60% V̇O_2peak_.

Parameter	PEC	DEC	*p* Value
Intensity (%V̇O_2peak_)	61.4 ± 14.4	59.8 ± 12.2	0.714
Heart rate (bpm)	112 ± 11	113 ± 16	0.823
V̇O_2_ (L/min)	1.06 ± 0.28	1.02 ± 0.35	0.377
V̇CO_2_ (L/min)	0.94 ± 0.23	0.87 ± 0.29	0.096
RER	0.89 ± 0.04	0.85 ± 0.02	0.019
O_2_ pulse (mL/min)	9.5 ± 2.3	9.0 ± 2.9	0.331
V̇E (L/min)	31.6 ± 8.4	30.2 ± 13.4	0.522
PETO_2_ (kPa)	14.6 ± 0.71	14.4 ± 0.72	0.102
PETCO_2_ (kPa)	4.8 ± 0.4	4.8 ± 0.6	0.984
EqO_2_	26.5 ± 3.8	25.4 ± 4.7	0.237
EqCO_2_	29.8 ± 3.4	29.7 ± 5.3	0.915
Carbohydrate oxidation (g/min)	0.80 ± 0.21	0.63 ± 0.19	0.009
Carbohydrate oxidation (% total energy)	64.1 ± 13.3	53.4 ± 7.5	0.060
Lipid oxidation (g/min)	0.21 ± 0.11	0.26 ± 0.11	0.084
Lipid oxidation (% total energy)	35.9 ± 13.3	46.6 ± 7.5	0.014
Total energy (kcals)	228.6 ± 57.9	216.6 ± 73.7	0.242

V̇O_2_: volume of oxygen uptake. V̇CO_2_: volume of carbon dioxide production. RER: respiratory exchange rate (ratio of V̇CO_2_ divided by V̇O_2_). O_2_ pulse: oxygen pulse (V̇O_2_/HR). V̇E: minute ventilation. PETO_2_: end-tidal pressure of oxygen. PETCO_2_: end-tidal pressure of carbon dioxide. EqO_2_: ventilatory equivalent for oxygen (≈V̇E/V̇O_2_). EqCO_2_: ventilatory equivalent for carbon dioxide (≈V̇E/V̇CO_2_).

## Data Availability

Data are contained within the article.
